# Classification and Feature Extraction Using Supervised and Unsupervised Machine Learning Approach for Broiler Woody Breast Myopathy Detection

**DOI:** 10.3390/foods11203270

**Published:** 2022-10-20

**Authors:** Aftab Siddique, Charles B. Herron, Jaroslav Valenta, Laura J. Garner, Ashish Gupta, Jason T. Sawyer, Amit Morey

**Affiliations:** 1 Department of Poultry Science, Auburn University, Auburn, AL 36849, USA; 2 Department of Animal Science, Czech University of Life Sciences Prague, 16500 Prague, Czech Republic; 3 Department of Business Analytics and Information, Auburn University, Auburn, AL 36849, USA; 4 Department of Animal Sciences, Auburn University, Auburn, AL 36849, USA

**Keywords:** bioelectrical impedance, hand palpation, in-line processing, supervised learning, unsupervised learning, woody breast

## Abstract

Bioelectrical impedance analysis (BIA) was established to quantify diverse cellular characteristics. This technique has been widely used in various species, such as fish, poultry, and humans for compositional analysis. This technology was limited to offline quality assurance/detection of woody breast (WB); however, inline technology that can be retrofitted on the conveyor belt would be more helpful to processors. Freshly deboned (*n* = 80) chicken breast fillets were collected from a local processor and analyzed by hand-palpation for different WB severity levels. Data collected from both BIA setups were subjected to supervised and unsupervised learning algorithms. The modified BIA showed better detection ability for regular fillets than the probe BIA setup. In the plate BIA setup, fillets were 80.00% for normal, 66.67% for moderate (data for mild and moderate merged), and 85.00% for severe WB. However, hand-held BIA showed 77.78, 85.71, and 88.89% for normal, moderate, and severe WB, respectively. Plate BIA setup is more effective in detecting WB myopathies and could be installed without slowing the processing line. Breast fillet detection on the processing line can be significantly improved using a modified automated plate BIA.

## 1. Introduction

**Figure 1:**
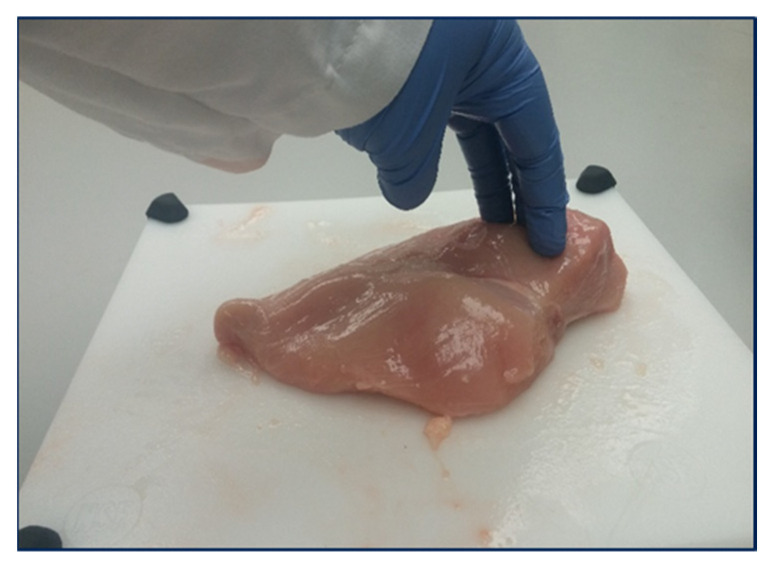
Hand palpation of fillets for manual classification based on severity level.

Poultry is a widely consumed form of protein in the United States, followed by beef and pork [1]. According to the National Chicken Council, approximately 113.58 lbs. of total poultry meat will be consumed per person by 2022, which is more than almost any other country [1]. Consumers primarily choose poultry meat because of the nutritional and functional characteristics, including consistency, appearance, and taste [2]. Additionally, markets are constantly shifting due to customer preferences and expectations, guiding the industry to produce greater volumes of trimmed chicken meat. Poultry breeders have boosted growth rates of birds through a combination of feeding programs, genetic selections, and improvement in animal husbandry practices to meet the growing market pressure for white meat. Consumer demand for improved quality poultry white meat has increased overall carcass output [3]. Nonetheless, fast-growing birds with greater breast meat yields have developed myopathies in their breast muscles, resulting in meat quality problems such as WB (Figure 1). According to Lui et al. [4], the global occurrence of WB is estimated at 20% and may be increasing. With many factors influencing meat quality, the most important factors focus on appearance, water retention capacity, color, and texture of the meat [5]. Fresh poultry meat characteristics can influence the sensory qualities or consuming behavior of a product and its acceptability by consumers [6].

## 2. Related Works

Woody breast (WB) is a myopathic condition that has plagued the broiler meat industry for years [7]. Woody breast in chicken breast fillets can be found at various levels of severity [8]. In a recent survey conducted by de Almeida Mallmann [9], moderate and severe WB increased by 10% (from 25 to 35%) in chicken breeds with increased yield, and a 5% increase was observed (25 to 30%) in mild WB fillets [7]. Barbut [10] estimated that severe WB instances are detrimental to the poultry industry resulting in annual losses of $200 million and estimated to reach $1 billion each year in North America [10]. Therefore, various systems have been implemented to identify, characterize, and classify chicken breast fillets based on WB myopathic severity [11,12,13,14,15]. To limit time and effort, inline non-destructive identification of these myopathic conditions is needed for both small- and large-scale poultry processors [16]. Several studies have evaluated different approaches for rapid and accurate identification of myopathic fillets including computer image recognition systems, near-infrared (NIR) spectrographic analysis, and a combination of low-coherence interferometry and hyperspectral imaging (HSI) [14,17,18,19]. The major advantage of these systems is that they can detect myopathy in fillets without contacting the fillets unlike BIA wherein the electrodes have to touch the meat. However, computer vision systems separate myopathic meat based on the image of the fillet, but analyzing the biochemical component of the meat would be a better predictor for detecting meat myopathies. Technologies such as HIS and NIR have shown promise as they analyze the biochemical characteristics of the breast fillets, but they require equipment with a large footprint and need complex data pre-processing prior to data analysis. The BIA technology used in the current study is a simple 4-electrode hand-held device that can be retrofitted in conveyor belts (as studied); the device has a versatile form factor, is easy to use and generates resistance and reactance data for myopathy fillet classification.

The primary working principle of BIA is based on the resistance property of a conducting material or wire, which is inversely proportional to its cross-sectional area and directly proportional to its length. However, the differences in material attributes, such as their form, shape, density, and composition, alter the rate of impulse conductance [16]. Conductivity of an electric current is determined not only by the physiological portion of the cytosol, but also by the frequency response used in the prediction. Thus, signals can shift due to minor changes in muscle anatomy [17,19,20]. Several recent exome sequencing, meta-proteomics, and proteomics investigations have discovered several variables such as oxidative stress and changes in intracellular calcium that may contribute to the development of WB [21,22,23,24,25].

Bioelectrical impedance analysis technology has been utilized in many species to determine physical composition and qualities, such as total body fluid content and fat content, by measuring electrical resistance or impedance. In the food industry, BIA parameters can be adjusted to specific species, and it has been utilized in fish for quick detection of proximate composition and pre-harvest conditions to improve fish quality [26,27,28,29]. Morey et al. [7] and Siddique et al. [30] have reported that BIA may be used to successfully detect WB fillets as an alternative to hand-palpation, reducing hand classification mistakes and increasing the accuracy of detecting WB.

Furthermore, BIA can detect other overlapping breast muscle myopathies, such as spaghetti meat, which is a myopathic condition impacting the chicken industry. Classification using BIA data can be improved using advanced data analytic technologies such as machine learning (ML), which includes data mining, artificial neural networks (ANN), deep learning (DL), and artificial intelligence [30]. Siddique et al. [30] reported that machine learning techniques such as Support Vector Machines (SVM) and backpropagation neural network (BPNN) classification algorithms could be another approach to classify the myopathic fillets based on severity levels [30].

**Figure 2:**
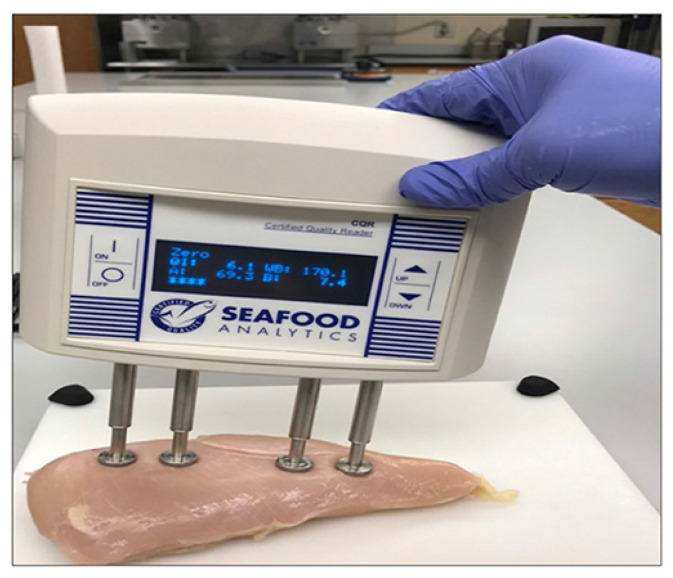
Hand-held BIA setup for the classification of fillets based on different myopathic conditions.

**Figure 3:**
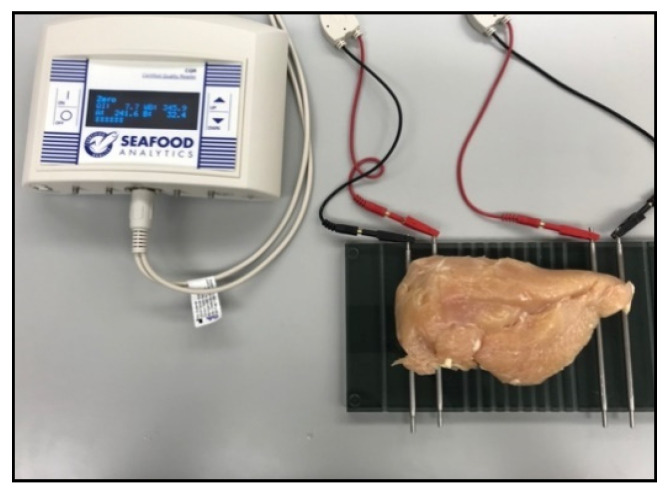
Plate BIA device for the classification of fillets based on severity level.

This research aims to assess the performance of two different bioelectrical impedance analysis (BIA) (Figure 2 and Figure 3) device setups as a prospective quantitative approach to detect WB fillets with differing severity degrees to develop an inline WB detection system. The presented article is divided into subsections on the detailed descriptions, materials, and methods used in Section 3; Section 4 describes the data analysis; Section 5 describes the results and discussion; and Section 6 concludes the research article.

## 3. Materials and Methods

For the proposed experiment, 56-day-old broiler (Ross 708) deboned chicken breast fillets (*n* = 80) were obtained from a commercial poultry processor and transported to the Department of Poultry Science at Auburn University. Chicken breast fillets were categorized (Figure 1) by an experienced team member based on WB severity (normal, moderate, and severe) using hand palpation [10]. During the hand-palpation technique, an experienced team member inspected each chicken breast fillet based on the perceived hardness in the three WB categories. The CQ Reader (Seafood Analytics, Version 3.0.0.3, Clinton Town, MI, USA) [27,28], with four spring-loaded electrodes (2 receiver probes and two electrical signals sending probes), was used to collect data from an inbuilt algorithm of the equipment (RJL Systems, Detroit, MI, USA). Four electrodes were inserted into the geometric center of each breast fillet along the ventral surface (Figure 2). The device then measured the response for fat indices and protein indices data, resistance, and reactance and retrieved the encrypted data for assessment (Seafood Analytics Certified Quality Reader, Version 3.0.0.3, Seafood Analytics, MI, USA). Weighing balance (OHAUS Corporation, Pine Brook, NJ, USA) was used to measure the weight of separate fillets, and the acquired data was used in the analysis.

### 3.1. K-Means Clustering (k-Means)

Clustering is a powerful approach to information retrieval and machine learning algorithms for predicting and summarizing data. It has been used effectively in diverse industries, including product differentiation, social platform analysis, document classification, and image classification in the food industry [31,32,33,34,35,36,37,38]. The aim of clustering is to put similar observations together and separate dissimilar ones [39].

**Figure 4:**
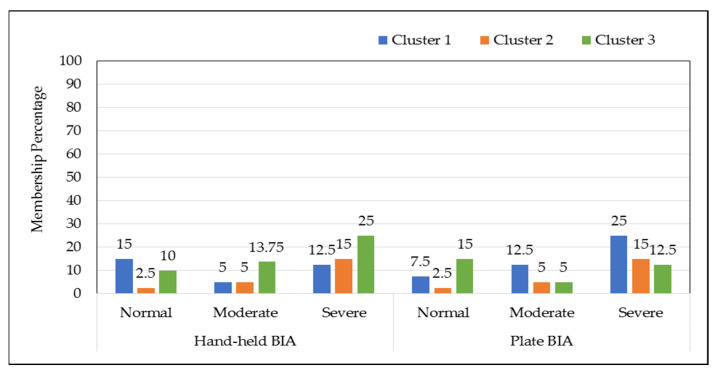
Membership cluster percentage formed in fuzzy c means analysis for different BIA setups.

Clustering algorithms are valuable for many applications, but their capabilities are severely constrained. If each observation is assigned to a single cluster, then the data is explained by all aspects of k and distinct clusters. As a rule of thumb, discontinuous data splitting is not always the most accurate way to represent it. It is possible to have numerous clusters in the same observation area [40]. The discontinuous segmentation is not always the best representation of the analyzed information because the data can have a significantly larger and more sophisticated hidden interpretation [41,42,43,44,45]. In a given data set, *k*-means algorithms (Figure 4) were applied to the points in a d-dimensional space in which *X* = {*x*_1_; *x*_2_; … *x_n_*}, in R^D^, i.e., N points (vectors) each with different D components in the data frame, partitional algorithms used in *k*-means clustering divides *X* into *k* full attributes, and mutually referenced clusters *P =* {*P*_1_, *P*_2_; …; *P_K_*}, $U_{i-1}^{k}P_{i}=X,\;P_{i}\cap P_{j}=0$ for 1≤i≠j≤K. This algorithm generates the clusters by optimizing function and the sum of squared error can be explained as [43,44]:

(1) $$SSE=\overset{k}{\underset{i=1}{\sum }}\underset{x_{j}\in P}{\sum }{\left||x_{j}-C_{i}|\right|}_{2}^{2}$$

where *||x_j_* − *c_i_||^2^* represents the Euclidean norm and *c_i_* = $1∕\left|P_{i}\right|\sum_{x_{j}\in P_{i}}x_{j}$ denotes the cluster centroid (*P_i_*), whose property of the data group is |*P_i_*|. The minimal optimization of the above equation is also well known as the problem of minimum sum of squared error (SSE) clustering. The resulting *k*-means algorithm clusters for each data point get clustered into “one and only one of the *k* partitions”. Data points with the same cluster-ID are in the same clusters and vise-versa [44]. For the initialization of *k*-means clustering algorithms, the initial *k* value is needed based on prior knowledge about the data, how many clusters are needed for the whole data set, and the number of clusters found by exploration data analysis (EDA) [44]. The *k*-means algorithms represented in Equation (1) start to cluster in a repetitive process in two alternative manners: (i) cluster-ID is assigned to each data point in the vector space, and (ii) updating the clusters as the data point changes in dimensional space. This process continues until there is no change in the data point position. The number of continuous repetitive steps may depend on vector points (N). Due to the linear comparison of data, the nature of *k*-means algorithms in the dimensional space also shows the linear dimensionality of the data. The working algorithm of the *k*-means cluster follows these steps:

Input: Data set (D) and initial cluster values depending on the prior knowledge, i.e., based on experience or exploratory data analysis. Output: Set a cluster representative *P* in the vector space.
Repeat: Assign the data points to the closest cluster mean and update the cluster ID of *j*th point in the data set.
Relocation of means: Update *P* so that *P_j_* is the mean of the *j*th cluster until the whole algorithm converges in Equation (1) with minimum local optima.

### 3.2. Fuzzy C Means (FCM)

Using fuzzy logic principles makes it possible to group highly dimensional data into clusters, assigning each point a percentage of membership in each cluster center between 0 and 100% [46]. Compared to typical hard-threshold clustering (*k*-means), in which every point is allocated a clear, accurate identity, FCM could provide improved clustering outcomes in certain types of datasets that do not lend themselves to traditional clustering approaches. This approach operates by assigning participation to each data point belonging to the nearest centroid on the basis of the distance between both the nearest cluster and the target value, calculated using a distance matrix [47]. The closer the data is to the nearest cluster, the greater the likelihood that it will belong to those specific centroids. The sum of each data point is entirely participation-based and should always be equal to one [48]. FCM is an unsupervised clustering approach used extensively for selecting features, clustering, and classifier designing challenges in astronomy, chemistry, geophysics, and medical diagnosis [49,50]. A clustering technique built on the Ruspini Fuzzy clustering concept was introduced in the 1980s due to the evolution of fuzzy theory [51].

This technique is used to analyze the data coordinates based on their distance from each other. For each grouping, the centroids are constructed depending on the spacing among sample points in the original data set. The developed algorithm for the fuzzy c-means clustering is as follows [49,50]:

Step 1. Calculate the center of the given data set

(2) $$v_{ij}=\overset{n}{\underset{k=1}{\sum }}{\left(u_{ik}\right)}^{m}x_{kj}/\overset{n}{\underset{k=1}{\sum }}{\left(u_{ij}\right)}^{m}\;$$

Step 2. Calculate the matrix distance (*D _[c,n]_*)

(3) Dij=∑j=1mxkj−vij212

Step 3. Updating of partition matrix for *n*th step as

(4) $$\;u_{ij}^{n-1}=\left(\frac{1}{\sum_{j-1}{\left(d_{ik}^{n}∕d_{jk}^{n}\right)}^{\frac{2}{m}-1}}\right)$$

Termination of fuzzy *c* means the algorithm only takes place when the algorithm reaches ||*U* ^(*k*+1)^ − *U* ^(*k*)^|| < *δ*; if not achieved, the algorithm returns to step 2 and re-executes until the conditions are being met by continuously updating the centroid [51].

### 3.3. K-Nearest Neighbor (k-NN)

The *k*-nearest neighbor (*k*-NN) technique was developed to perform statistical techniques when valid parametric estimates of probability densities are unknown or difficult to calculate. Fix and Hodges [52] presented a non-parametric design data classification approach in 1951 through an unpublished paper by the US Air Force School of Aviation Medicine, the *k*-nearest neighbor rule [52], which was further developed by Thomas Cover [53] in 1967. A new observation is classified depending on how comparable it is to other observations already analyzed [54]. According to its neighboring labels, this classification is performed. Various industries have applied *k*-NN, including cyber security, information security, and aviation. Valuable life forecast, defect categorization, nephropathy diagnosis in children, and infiltration prevention systems have been implemented using the *k*-NN method [55,56,57,58].

For understanding the concept and working principle of *k*-NN, let us suppose that we have a data set for different conditioned myopathic chicken breast fillets representing resistance and reactance (Supplementary file, Figure S2, blue points represent resistance and red points represents reactance). Now suppose that we added a new data point for resistance and reactance and were told that the new fillets’ value in the dataset in the class is a severe woody breast (value represented by square box, Figure 2). Let’s see if the *k*-NN algorithm is able to identify the class by input data. For the classification of a new data point, the *k*-NN general rule must be followed as [51,52,53,54].

Step 1. Input: *V*, *V_l_*, *c*, i.e., *V* = training data set, *V_l_* = labels of data set, *c* = sample data point that needs to be classified.

Step 2. For v to training data set size: Compute the distance between the training data set and sample data point d (*V_i_*, *c*).

Step 3. End for loop: Select the desired number of clusters of nearest neighbors, arrange the computed distance in increasing order, and count the number of occurrences for each label in the top *k*-neighbors.

Step 4. Output: Assign c to the most occurring label (*l*).

The *k*-nearest-neighbor classifier is based on the Euclidean distance between a test sample and the specified training samples. The Euclidean distance between sample *V_i_* and *V_l_* (*l* = 1, 2, …, *n*) is defined as *d* (*V_i_*, *V_l_*) = √(*V_i_*_1_ − *V_l_*_1_)^2^ + (*V_i_*_2_ − *V_l_*_2_)^2^ + ⋯+ (*V_ip_* − *V_l__N_*)^2^ [51]. After the computation of the new data set and evaluation of distance between the original training data set and test dataset, we can decide based on the neighbors where the new data point belongs (Supplementary file, Figure S2). In Supplementary file, Figure S2, the new observation is classified as severe fillets with minimum numbers of *k*-neighbors (*k* = 2). For *k* = 2, closest neighbors are in a small circle. It can be clearly observed that if *k* = 2, two of the neighbors are severe, so the new data point would also be classified as severe. So, for the classification of a new data point added into the data set, it will get classified as severe fillet. However, this does not imply that *k* = 2 is the highest performance quantity for the dataset; more additional observations should be classified with other *k* values to identify the quantity with the optimum overall performance from the data set.

### 3.4. Support Vector Machines (SVM) Algorithms

Vapnik [58] proposed the SVM method for the first time in 1995, and it has received a tremendous amount of attention from the machine learning applications community. Many studies have found that the SVM approach outperforms other data classification algorithms depending on the data type regarding classification accuracy compared with other methods [59,60]. For data set categorization, SVM generates a line between two or more classes, referred to as hyper-planes. SVM aims to discover a hyperplane that can separate two classes of provided data with a maximum margin while still offering the best generalization capability for a two-class linearly separable learning assignment. It provides highly accurate results on the training dataset and high predicting accuracy for the new dataset from the same population as the training dataset [30]. A detailed working principle for the SVM algorithm can be found in Siddique et al. [30].

### 3.5. Bioelectrical Impedance Analysis

Samples collected from local processors were placed on a non-conducting surface for BIA analysis. The resistance and reactance qualities of myopathic conditioned fillets were measured with a hand-held BIA device and Plate BIA device (Seafood Analytics, Clinton Town, MI, USA) on the upper surface of the chicken breast fillet. Both BIA units are made up of four electrodes: two signal electrodes and two detecting electrodes. These electrodes are connected to an AC current of 800 µA and 50 kHz, and they are able to produce voltage fluctuations ranging from 3.75 to 10.60 V [7]. The electrodes used in the collection of data were made of stainless steel and are used to complete the circuit between the chicken breast fillets and the four electrodes (RJL Systems, Detroit, MI, USA). In hand-held BIA, the device was held in hand to perform compression-based analysis (Figure 2) whereas samples were placed on the plate BIA to collect the data (Figure 3). As soon as the electrodes come into contact with the item being tested, the circuit is completed, and the instrument begins to take two sets of measurements.

## 4. Data Analysis

Data were analyzed using two different BIA configurations (Hand-held and Plate setup) for a descriptive summary of the collected values on the plate BIA of Resistance, Reactance, and Breast fillet weight using SAS software (Version 9.4, Cary, NC, USA) and Tukey HSD means value. Data collected from both BIA setups were subjected to R Software and jmp16 Pro (Version 16.0, Cary, NC, USA) by implementing the unsupervised (*k*-means clustering, fuzzy c-means clustering) and supervised learning algorithms (*k*-nearest neighbor, SVM) to evaluate the accuracy of the equipment for the detection of WB in chicken breast fillets. The clustering method was implemented in our data set to observe the identification efficiency of both BIA methods in the classification of myopathic fillets without using labels or any supervision. The collected data used in the analysis had 3 dimensions, i.e., resistance, reactance, and weight of individual fillets. For *k*-means and FCM analysis for the BIA collected data, R software (Version 4.2.0, Vigorous Calisthenics, Auckland, New Zealand) was used with set replicability of 3000 using the set seed command. For the development of supervised learning algorithms using *k*-NN models, the data were randomly divided into 55:45 training and testing sets using validation options in jmp 16 Pro. For the SVM analysis of the collected data, the caret package was used in R software (Version 4.2.0, Vigorous Calisthenics, Auckland, New Zealand). The caret package algorithm calculated the best-suited tuning parameter or cost (C) for both the training and testing data sets. A seed value (replicability) was set at 3000 for the SVM analysis. 

## 5. Results and Discussion

Table 1 illustrates the differences in multiple factors determined by different BIA setups among the different severity categories of WB meat. In Table 1, there are statistically significant differences (*p* < 0.05) in the resistance and reactance for normal chicken breast fillets and moderate breast fillets, but there were no significant differences observed between normal fillets and severe WB fillets in hand-held BIA collected data (Table 1). Our results for the plate BIA and hand-held BIA setup agree with the study conducted and reported by Morey et al. [7], in which the authors have found a lower resistance and reactance value for normal chicken breast fillets as compared with severe chicken breast fillets, and vice-versa. Reactance and resistance are affected by the compositional content of a product. They are measurements of a substance’s capacity to accommodate a charge and carry or conduct electrical charges, respectively [61]. Histological changes in growing birds can alter the water distribution within the muscle structure, which influences or changes the electrical properties of chicken breast fillets [19]. In our presented data, there were no statistically significant differences observed in resistance and reactance values for normal chicken breast filets (72.89 Ω and 25.76 Ω, respectively) compared with severe WB filets (70.60 Ω and 21.76 Ω) for the hand-held BIA collected data.

On the other hand, data collected from the plate BIA showed that normal chicken breast fillets have a resistance of 103.34 Ω and severe WB fillets have an average resistance of 112.02 Ω. Kyle et al. [19] have mentioned that at lower frequencies, muscles and their other components, such as fatty tissues and peptide residues, act as an insulator and do not allow the flow of electrical charges, which in results forces these electrical charges to flow from alternative components of the cell, i.e., extracellular fluid. The authors furthermentioned that increasing non-conducting suspended elements in water will also increase the resistance capacity of the fluid system. Severe WB fillets have a high amount of extracellular fluid. They have these non-conducting suspended components, which ultimately increase the resistance of WB fillets as compared with normal chicken breast fillets [7,30,62,63].

The classification experiment was conducted on two different BIA configurations generating data sets for WB fillets classified as normal, moderate, and severe. In the *k*-means clustering algorithm (unsupervised method), three clusters were formed using the resistance, reactance, and weight of fillets. The *k*-means clustering results for the plate BIA data showed that the average distance value of each observation from the center of clusters one, two, and three ranged from 0.14 to 2.50 for normal fillets, 0.45 to 3.83 for moderate fillets, and 0.46 to 5.09 for severe fillets (Supplementary file, Figure S1). The first cluster means for the plate BIA method for resistance, reactance, and weight were 101.25 ± 13.13 Ω, 27.06 ± 5.67 Ω, and 426.68 ± 44.17 gm, respectively; the second cluster means for resistance, reactance, and weight were 109.62 ± 11.66 Ω, 34.66 ± 6.71 Ω, and 556 ± 44.84 gm, respectively; the third cluster means for resistance, reactance, and weight were 143.21 ± 8.73 Ω, 56.25 ± 5.21 Ω, and 542.28 ± 67.95 gm, respectively (Supplementary file, Table S1).

The hand-held BIA cluster data classification showed that the average distance value of each observation from the center of clusters one, two, and three ranged from 0.04 to 4.73 for normal fillets, 0.07 to 2.22 for moderate, and 0.20 to 7.49 for severe fillets, respectively. The first cluster means for the hand-held BIA method for resistance, reactance, and weight were 64.52 ± 4.88, 19.78 ± 4.71, and 571.36 ± 38.29, respectively; the second cluster means for resistance, reactance, and weight were 81.71 ± 5.91 Ω, 31.80 ± 3.33 Ω, and 444.12 ± 52.44 gm, respectively; the third cluster means for resistance, reactance, and weight were 70.75 ± 3.04 Ω, 21.63 ± 4.47 Ω, and 475.26 ± 33.10 gm, respectively (Supplementary file, Table S2). Generally, average distance values in clustering are not very informative but they can provide us with an idea of how much the clusters are separated from each other. The more the average distance between the clusters the better is the clustering results. In our presented data, the *k*-means clustering results for hand-held BIA showed a better average distance value for clusters of normal fillets and severe fillets as compared with the data collected for the plate BIA. Data obtained from the plate BIA indicates that 18.75% normal, 13.75% moderate, and 22.50% severe WB fillets were placed in cluster 1; 6.25% normal, 7.50% moderate, and 23.75% severe fillets were placed in cluster 2; and 1.25% normal, 0.00% moderate, and 6.25% severe fillets were placed in cluster 3.

However, data obtained for hand-held BIA concluded that 2.50% normal, 6.25% moderate, and 26.25% severe WB fillets were clustered in cluster 1; 8.75% normal, 2.50% moderate, and 6.25% severe WB fillets were clustered in cluster 2; 15.00% normal, 12.50% moderate, and 18.75% severe fillets were clustered in cluster 3 (Figure 4). Based on the optimal number of clusters (*k* = 3) needed for better clustering average Silhouette Index value for plate BIA and hand-held BIA was 0.7688 and 0.8036, respectively. Indicating that, *k*-means clustering analysis performed on the BIA data set creates dense and compact clusters with less overlapping between cluster data points. Separate and compact clusters clearly indicated that both BIA devices are capable of identifying myopathic conditions in non-labeled data with some degree of overlapping.

Fuzzy c means membership analysis showed that the hand-held BIA data for chicken breast fillets were clustered as 15% normal, 5% moderate, and 12.5% severe in cluster 1; 2.5% normal, 5% moderate, and 15% severe in cluster 2; and 10% normal, 13.75% moderate, and 25% severe in cluster 3 compared with the plate BIA data in which chicken breast fillets were clustered as 7.5% normal, 12.5% moderate, and 25% severe in cluster 1; 2.5% normal, 5% moderate, and 15% severe in cluster 2; and 15% normal, 5% moderate, and 12.5% severe in cluster 3 (Figure 4). The data analyzed for hand-held BIA showed that the probability percentage of normal fillets in cluster 1 to cluster 3 ranges from 9.09% to 54.5%; probability for moderate fillets in cluster 1, 2, and 3 ranges from 21.0% to 57.8%; and for severe fillets in the three distinct clusters ranged from 23.8% to 47.6%. On the other hand, the plate BIA analyzed data for FCM clustering showed that the probability percentage for normal, moderate and severe conditioned myopathic fillets ranges from 10% to 60% (Table 2). Fuzzy c means is a soft clustering method that keeps all the observation values in each cluster, although they belong to different clusters at the same time. The hand-held BIA collected data showed much better separated clusters based on the Dunn’s coefficient value compared to those of the plate BIA collected value. The Dunn’s coefficient value for the hand-held BIA data was 0.775, and for the plate BIA data 0.741. Clusters formed by the hand-held BIA dataset (Supplementary file, Figure S3) showed better cluster formation than those of the plate BIA data (Supplementary file, Figure S4). The cluster achieved reduction for the hand-held BIA observation was 82.08%, and for the plate BIA data 77.87%. The hand-held BIA data showed a better fuzzy Silhouette Index value at *k* = 3 (as compared with the plate BIA data (Hand-held BIA fuzzy Silhouette Index value = 0.803 and Plate BIA fuzzy Silhouette Index value = 0.768) (Supplementary file, Figures S5 and S6).

The *k*-NN is an occurrence-based classification algorithm that distinguishes a new data point by analyzing it with an already stored value in the system which has been employed as the training set to analyze it with a screening set value. As indicated in Table 3, *k*-NN successfully classified both BIA data sets with superior efficiency in the testing set despite the overlapping resistance, reactance, and fillets weight data displayed in density (Supplementary file, Figures S7–S9). The *k*-NN analysis for the hand-held BIA setup showed better classification performance based on instance in the testing data set (normal = 50%, moderate = 42.90%, and severe = 87.50%) especially in comparison with the training data set (normal = 38.50%, moderate = 10.0%, and severe = 67.70%), and for plate BIA the categorization efficiency for the testing set was 40.0% for normal, 25% for moderate, and 78.60% for severe fillets. Both classification efficiencies were reported in the *k*-NN algorithm. The trained model was observed to be efficient in classification during the testing stage. Results obtained by training accuracy of both BIA methods for *k*-NN classification were 38.50–31.30% (Hand-held BIA-Plate BIA) for normal fillets, 10.00–7.71% (Hand-held BIA-Plate BIA) for moderate, and 57.70–57.10% (Hand-held BIA-Plate BIA) for severe fillets classification (Table 3), respectively, using the BIA parameters and fillet weight data set (*n* = 80). The testing set was found to be higher in accuracy as compared with our training set with 40.00–50.00% (Plate BIA-hand-held BIA) normal classified, 25.00–42.00% (Plate BIA-hand-held BIA) moderate classified, and 78.60–87.50% (Plate BIA Hand-held BIA) severe WB classified (*n* = 80; Table 3). The testing set data was higher in accuracy than the training set data, possibly due to the nature of myopathic fillets, i.e., presence of extracellular fluid in severe WB fillets and also the nature of the algorithm used. The *k*-NN algorithm, which is completely based on the instance-based counting method for the number of observations used in the training set and then assigning the next upcoming value-based to a most voted group called neighbors. A higher classification was observed in the BIA setup for the *k*-NN algorithm for the testing. Normal fillets showed 50.00%, moderate fillets 42.90%, and severe fillets 87.50% classification than in the training set classification labels (Table 3).

The results obtained from the SVM classification algorithm showed that the percent accuracy for the classification of normal WB fillets in the test data set was 80.00%; for moderate fillet classification, the testing set showed an accuracy of 66.67%; and the classification accuracy for the severe WB fillets for the test data set was 85.00% (Table 4). On the other hand, the data collected for the hand-held BIA system, analyzed using SVM analysis, showed the classification efficiency in the testing data sets for normal fillets was 77.78%, for moderate fillets 85.71%, and severe fillets 88.89% respectively (Table 4). Due to the nature of the uneven random distribution of data points in the training and testing split method, we also performed the *k*-fold cross validation technique to determine the overall accuracy of the developed SVM model for two different BIA datasets. Our results indicated that at the cost function of *C* = 1.5, with repeated cross-validation accuracy, for the hand-held BIA data was 92.05% (Supplementary file, Figure S10); while for the plate BIA it was 93.11% (Supplementary file, Figure S11) with the cost function of *C* = 0.5. The Confusion matrix table showed the number of test fillets correctly classified based on the labels (Supplementary file, Table S3). A total of 34 fillets were randomly selected for the testing data set, which includes 9 normal fillets, 7 moderate fillets, and 18 severe WB fillets in hand-held BIA data; while 6 normal fillets, 9 moderate fillets, and 20 severe WB fillets were selected for the plate BIA data. The Confusion matrix table (Supplementary file, Table S3) shows that from the hand-held BIA testing data set, two fillets were misclassified from the normal category, one fillet from the moderate condition was misclassified in the normal category, and two fillets from the severe WB condition in the normal and moderate category. In the plate BIA collected data, one fillet was misclassified in the moderate category; for moderate fillets, two fillets were classified under severe fillets, and one fillet was classified in the normal category. For severe WB fillets, 3 fillets were misclassified in the normal category.

Based on the analyzed data for the hand-held BIA and plate BIA, the sensitivity of normal, moderate, and severe fillets was 100%, 80%, and 100% respectively, i.e., if a breast fillet had a diagnosis of normal fillets, the probability that this myopathic fillet was correctly assigned to the normal category was 1.0. The specificity of the normal fillets classification was 94.7%, moderate 100%, and for severe fillets 100%, implying that 94.7% of the normal fillets were correctly classified as normal on the basis of their collected parameters, such as resistance and reactance. The results obtained in this study are in agreement with Siddique et al. [29], where the researchers found that the classification efficiency of the SVM algorithm in the categorization effectiveness of SVM for the partitioning of high dimensionality data showed improved classification efficiency for normal (training performance 63.86%, testing performance 71.04%), moderate (training performance 49.88%, testing performance 59.99%), and severe WB (training performance 49.88%, testing performance 59.99%). The authors have also found that the results for the overall repeated cross-validation accuracy percentage for the hand-held BIA and plate BIA were above 90%, which agrees with the study published by Geronimo et al. [15], where the authors reported the accuracy percentage of the SVM model as over 90%. The lower cost function of the plate BIA collected data in conjunction with the SVM model implies that the developed model can be easily implemented in the real-world scenario without much pre-processing of data. The architecture of the plate BIA plays a significant role in the collection and accuracy of data [20]. Long separate probes in the plate BIA setup allows the flow of electrical impulse throughout the fillets, while the hand-held probe BIA covers less area on the fillets’ surface. Additionally, Siddique et al. [29] found that conventional classification analysis methods such as LDA did not perform as well with the hand-held BIA setup, with an accuracy of 61.70% for normal chicken breast fillets, 31.30% for moderate WB fillets, and 68.50% for severe WB fillets in the training set, compared with 75.60% normal, 33.33% moderate, and 56.30% for severe WB in the testing data set [29]. When kernel functions are used in SVMs, the original input variables become axially separable in the higher-dimensional domain, allowing them to be classified. Furthermore, SVMs can reduce both the estimating dimension and error of the system simultaneously [31].

## 6. Conclusions

The results from the current work demonstrated that using a plate BIA in real-time inline chicken production systems to classify chicken breast fillets, depending on the severity of myopathy, is conceivable. Plate BIA detection for muscle histopathologic classification is superior to a hand-held BIA device when used in conjunction with SVM and *k*-nearest neighbor analysis. Input variables can provide a more efficient separation capability than the hand palpation method or other unsupervised algorithms used in BIA collected data for evaluating chicken breast fillets. In terms of the rapid detection of woody breast myopathies in chicken breast fillets without having pre-processing steps for data analysis is an important part of our research. For the collection and analysis of our data, no data pre-processing steps were involved for the clustering analysis using FCM and *k*-means. The results obtained in supervised learning techniques follow the same pattern of accuracy with an overall accuracy of 90% as that reported in previous research conducted using NIR, image analysis, and HSI imaging techniques where intensive pre-data processing is needed before the classification of woody breast fillets can be performed. The overall cross-validation accuracy with a lower cost function indicates that the developed SVM model for the plate BIA setup can be directly implemented in an actual processing plant for classification of fillets without implementing intensive data pre-processing steps.

Future studies are needed to collect and classify larger volumes of data on myopathic breast fillet for conditions such as white stripping, spaghetti meat condition, fillet time, processed bird age, different variations in used frequencies, and fillet temperature throughout the processing procedures. In addition, the use of different variables avoids overlapping circumstances induced by human counterparts during the inline processing of the fillets. It is apparent that through use of this novel technology chicken processing efficiency and classification of chicken breast fillets with undesirable WB myopathy can be improved.

## Figures and Tables

**Table 1:** Summary table for two different bioelectrical impedance device setups for resistance, reactance and fillet weights among woody chicken breast fillets with varying severity levels.

		Hand-Held BIA		Plate BIA	
WB Type	Fillet Weight (g)	Resistance (R; Ω)	Reactance (X_c_, Ω)	Resistance (R; Ω)	Reactance (X_c_, Ω)
Normal	473.54 ± 54.56 ^b^	72.89 ± 6.25 ^a^	25.76 ± 5.50 ^a^	103.34 ± 15.89 ^ab^	31.02 ± 8.91 ^a^
Moderate	510.10 ± 57.26 ^ab^	67.88 ± 6.19 ^b^	22.14 ± 5.51 ^b^	101.61 ± 14.50 ^b^	28.78 ± 9.02 ^a^
Severe	514.96 ± 69.33 ^a^	70.60 ± 8.24 ^ab^	21.76 ± 6.48 ^b^	112.02 ± 16.68 ^a^	33.98 ± 10.47 ^a^

^a^,^b^, Means with different superscript in columns are significantly different (*p* < 0.05) from each other.

**Table 2:** Percentage probability of fillets grouped into three different cluster.

Fillets Type	Cluster Probability Percentage for Hand-Held BIA			Cluster Probability Percentage for Plate BIA		
	Cluster 1	Cluster 2	Cluster 3	Cluster 1	Cluster 2	Cluster 3
Normal	54.5	9.09	36.3	30	10	60
Moderate	21.0	21.0	57.8	55.5	22.2	22.2
Severe	23.8	28.5	47.6	47.6	28.5	23.8

**Table 3:** *K*-nearest neighbor classification table for hand-held BIA, and Plate BIA collected parameters occurred with different severity levels of woody breast myopathies.

		Hand-Held BIA		Plate BIA	
WB Classification	No. of Fillets	Training (%)	Testing (%)	Training (%)	Testing (%)
**Normal**	21	38.50	**50.00**	31.30	**40.00**
**Moderate**	17	10.0	**42.90**	7.70	**25.00**
**Severe**	42	57.70	**87.50**	57.10	**78.60**

**Table 4:** Summary table of classification accuracies for hand-held BIA and plate BIA setup for normal, moderate, and severe woody chicken breast fillets using SVM algorithms.

		Hand-Held BIA		Plate BIA	
WB Classification	No. of Fillets	Testing (%)	No. of Classified Fillets/Total fillets	Testing (%)	No. of Classified Fillets/Total Fillets
**Normal**	21	**77.78**	**7/9**	**80.00**	**4/5**
**Moderate**	17	**85.71**	**6/7**	**66.67**	**6/9**
**Severe**	42	**88.89**	**16/18**	**85.00**	**17/20**

No. of classified fillets and total number of fillets are based on the testing data set.

## Data Availability

Not applicable.

## Supplementary Materials

The following supporting information can be downloaded at: https://www.mdpi.com/article/10.3390/foods11203270/s1, Table S1: Summary table for the average cluster distance values from the center of the clusters for Hand-held BIA and Plate BIA; Table S2: *K*-means clustering table for average cluster means for 3 different clusters for conventional BIA and plate BIA collected parameters occurred with woody breast myopathies; Table S3: Confusion matrix table for number of fillets classified in each labeled categories in testing data set using split data set method; Figure S1: Diagrammatic representation of *k*-means clustering; Figure S2: Diagrammatic representation of steps in *k*- nearest neighbor clustering analysis; Figure S3: Different clusters formed for hand-held BIA data collected for different severity level of fillets; Figure S4: Different clusters formed for plate BIA data collected for different severity level of fillets; Figure S5: Graph for optimal number of clusters based on Silhouette Index value for handheld BIA dataset; Figure S6: Graph for optimal number of clusters based on Silhouette Index value for plate BIA dataset; Figure S7: Density graph representing average distribution and overlapping of resistance (A) based on different severity levels; Figure S8: Density graph representing average distribution and overlapping of reactance (B) based on different severity levels; Figure S9: Density graph representing average distribution and overlapping of fillets weights (Wt) based on different severity levels; Figure S10: Hand-held BIA collected data graph for repeated cross validation accuracy for SVM model with cost function; Figure S11: Repeated cross validation accuracy graph for plate BIA collected data in SVM model with cost function.
