# Supplementary material for: Classification and Feature Extraction Using Supervised and Unsupervised Machine Learning Approach for Broiler Woody Breast Myopathy Detection
Source: Foods. 2022 Oct 20;11(20):3270. doi: 10.3390/foods11203270 (PMC9601423; doi:10.3390/foods11203270)
Supplement: Supplementary file 1 [file foods-11-03270-s001.zip › foods-1905159-supplementary.pdf]

Article

Classification and Feature extraction Using Supervised and Unsupervised Machine Learning Approach for Broiler Woody Breast Myopathies Detection

Aftab Siddique [1], Charles B. Herron [1], Jaroslav Valenta [2], Laura J. Garner [1], Ashish Gupta [3], Jason T. Sawyer [4] and Amit Morey [1,*]

[1] Department of Poultry Science, Auburn University, Auburn, AL 36849, USA
[2] Department of Animal Science, Czech University of Life Sciences Prague, 16500 Prague, Czechia
[3] Department of Business analytics and Information, Auburn University, AL 36849, USA
[4] Department of Animal Sciences, Auburn University, Auburn, AL 36849, USA
* Correspondence: azm0011@auburn.edu; Tel: +1-229-395-9837

All authors have read and agreed to the published version of the manuscript.

Supplementary File:

Table S1. Summary table for the average cluster distance values from the center of the clusters for Hand-held BIA and Plate BIA

WB Type	Cluster Number	Average distance range for Plate BIA	Average distance range for Hand-held BIA
Normal	1	0.14-2.50	0.04-4.73
Moderate	2	0.45-3.83	0.07-2.22
Severe	3	0.46-5.09	0.20-7.49

Table S2. K-means clustering table for average cluster means for 3 different clusters for conventional BIA and plate BIA collected parameters occurred with woody breast myopathies.

	Hand-held BIA			Plate BIA		
Clusters	Resistance Means ±SD	Reactance Means ±SD	Weight Means ±SD	Resistance Means ± SD	Reactance Means ± SD	Weight Means ± SD
1	64.52±4.88	19.78±4.71	571.36±38.29	101.25±13.13	27.06±5.67	462.68±44.17
2	81.71±5.91	31.80±3.33	444.12±52.44	109.62±11.66	34.66±6.71	556.27±44.84
3	70.75±3.04	21.63±4.47	475.26±33.10	143.21±8.73	56.25±5.21	542.28±67.95

Table S3. Confusion matrix table for number of fillets classified in each labeled categories in testing data set using split data set method

Fillets Type	Hand-held BIA			Plate BIA		
	Normal	Moderate	Severe	Normal	Moderate	Severe
Normal	7	1	1	4	1	0
Moderate	1	6	0	1	6	2
Severe	1	1	16	3	0	17

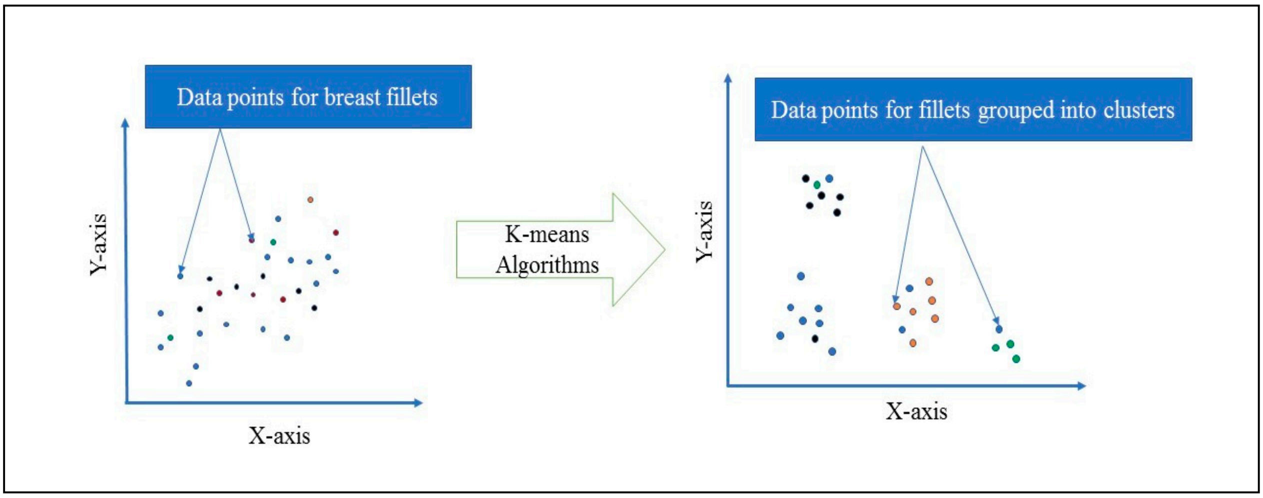

Figure S1. Diagrammatic representation of k-means clustering

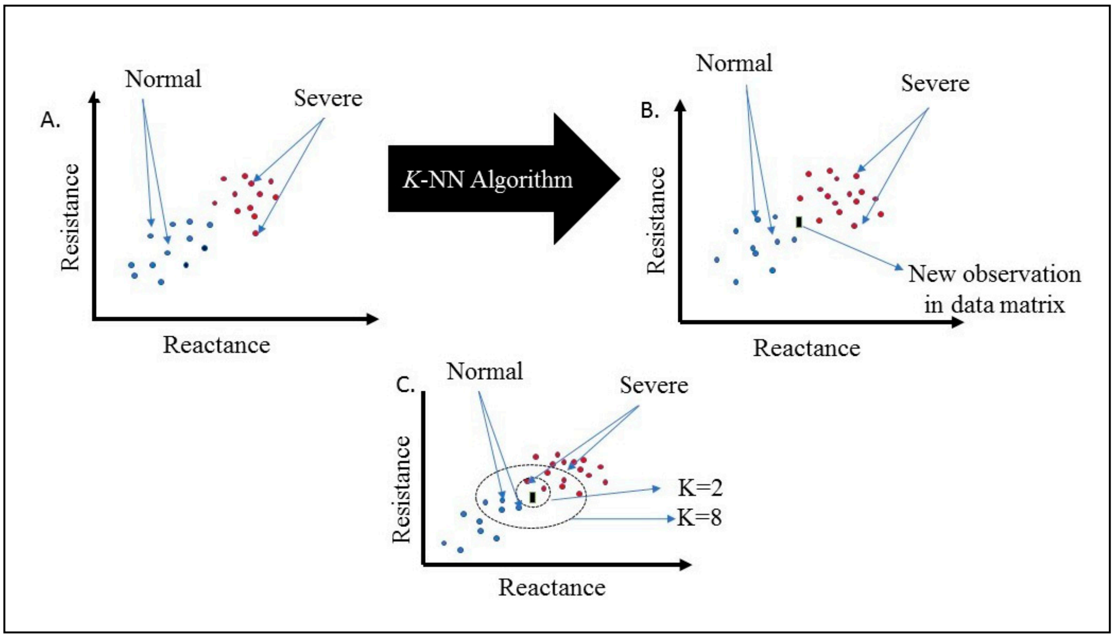

Figure S2. Diagrammatic representation of steps in *k*- nearest neighbor clustering analysis

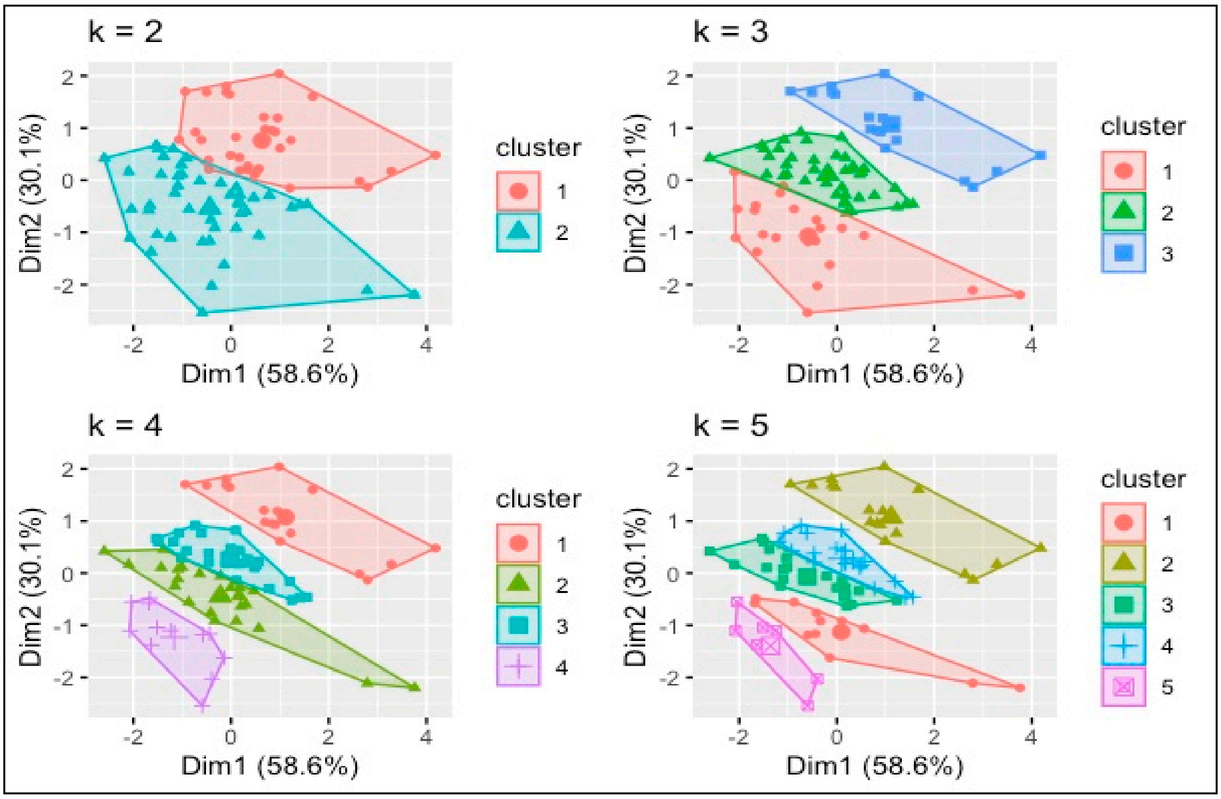

Figure S3. Different clusters formed for hand-held BIA data collected for different severity level of fillets

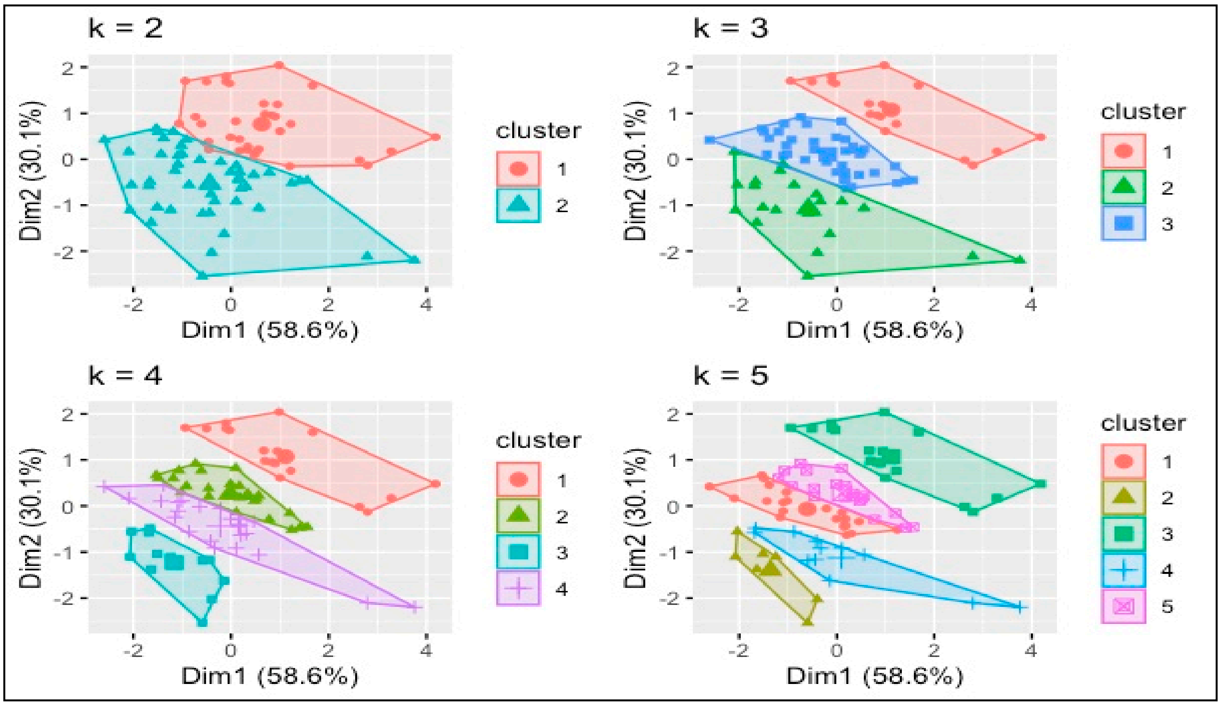

Figure S4. Different clusters formed for plate BIA data collected for different severity level of fillets

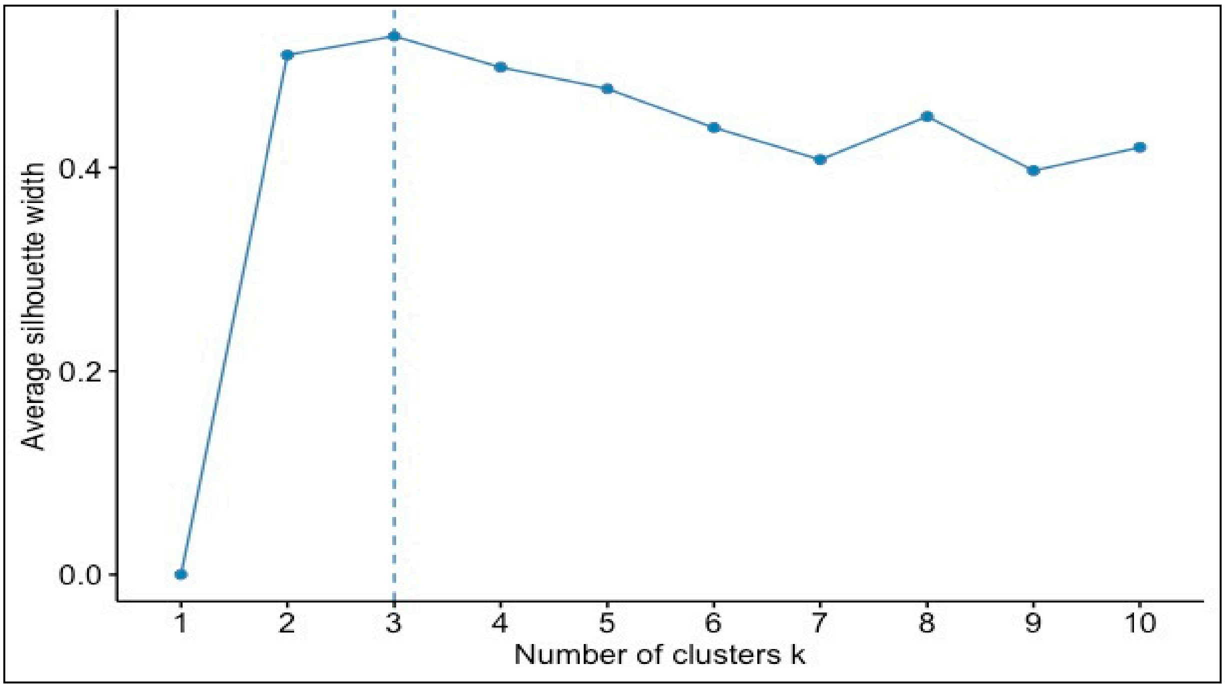

Figure S5. Graph for optimal number of clusters based on Silhouette Index value for handheld BIA dataset.

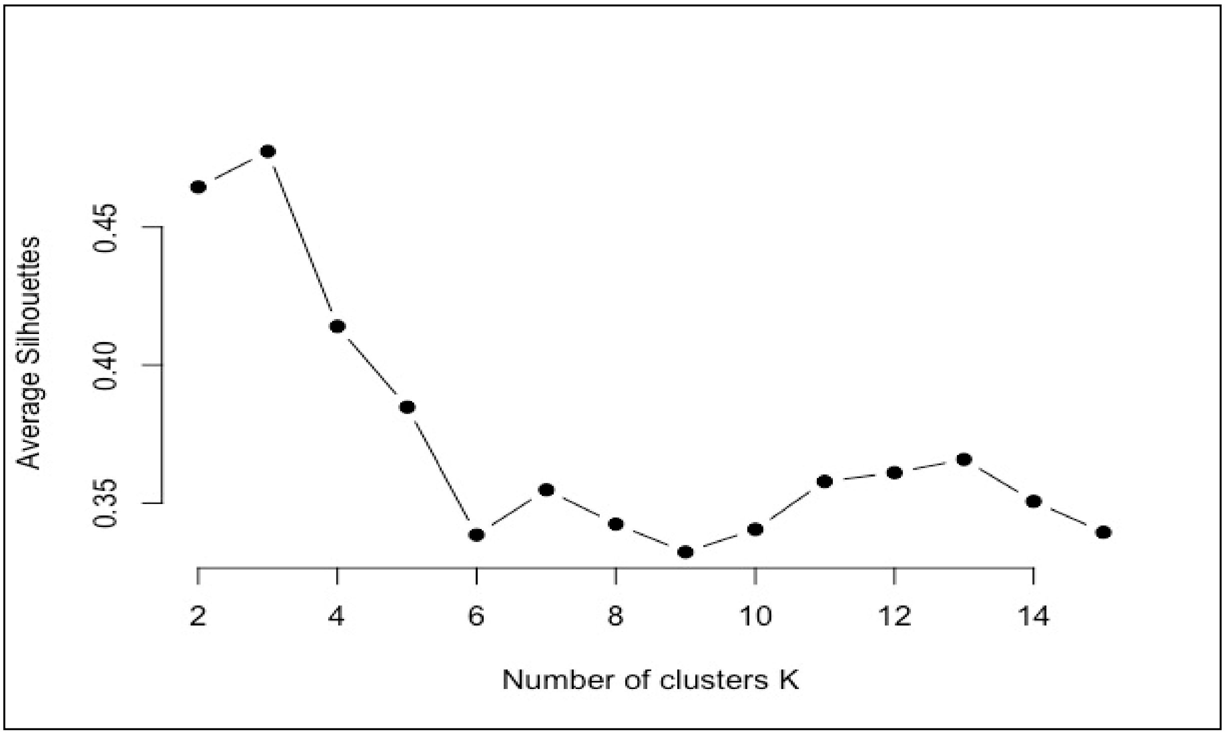

Figure S6. Graph for optimal number of clusters based on Silhouette Index value for plate BIA dataset.

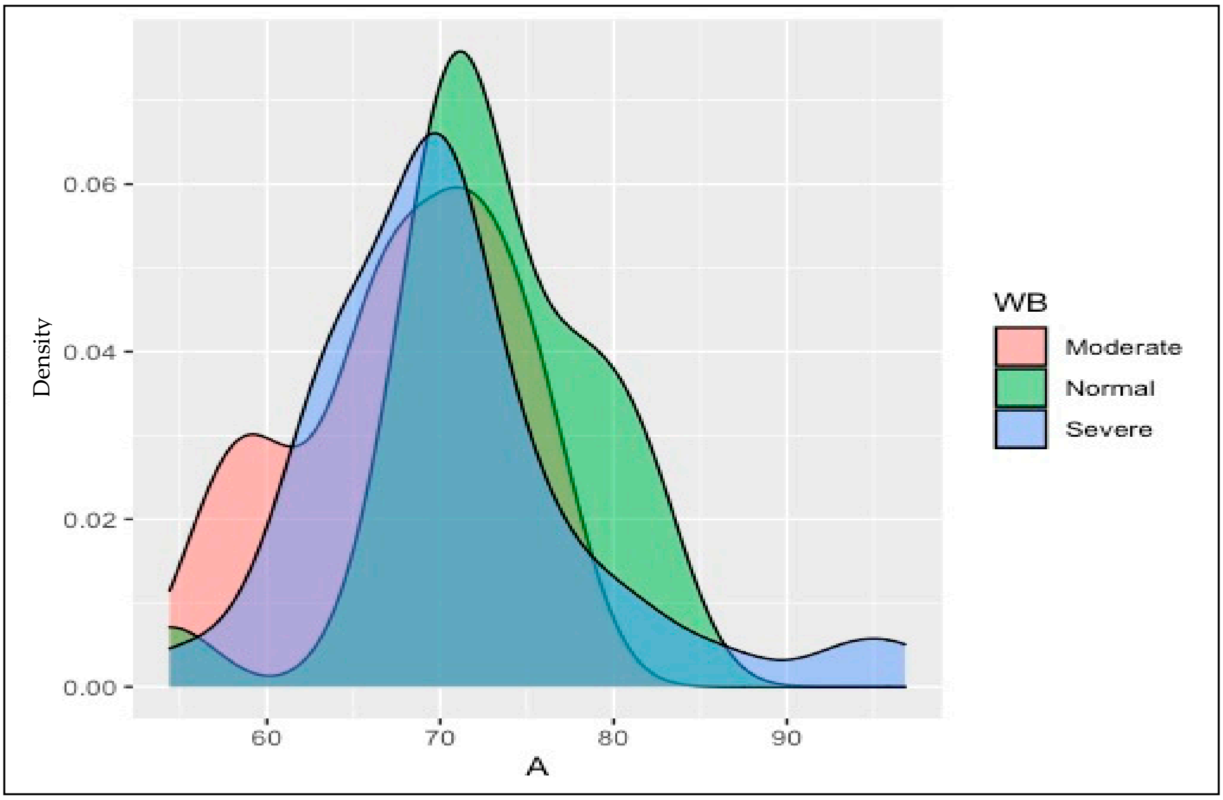

Figure S7. Density graph representing average distribution and overlapping of resistance (A) based on different severity levels

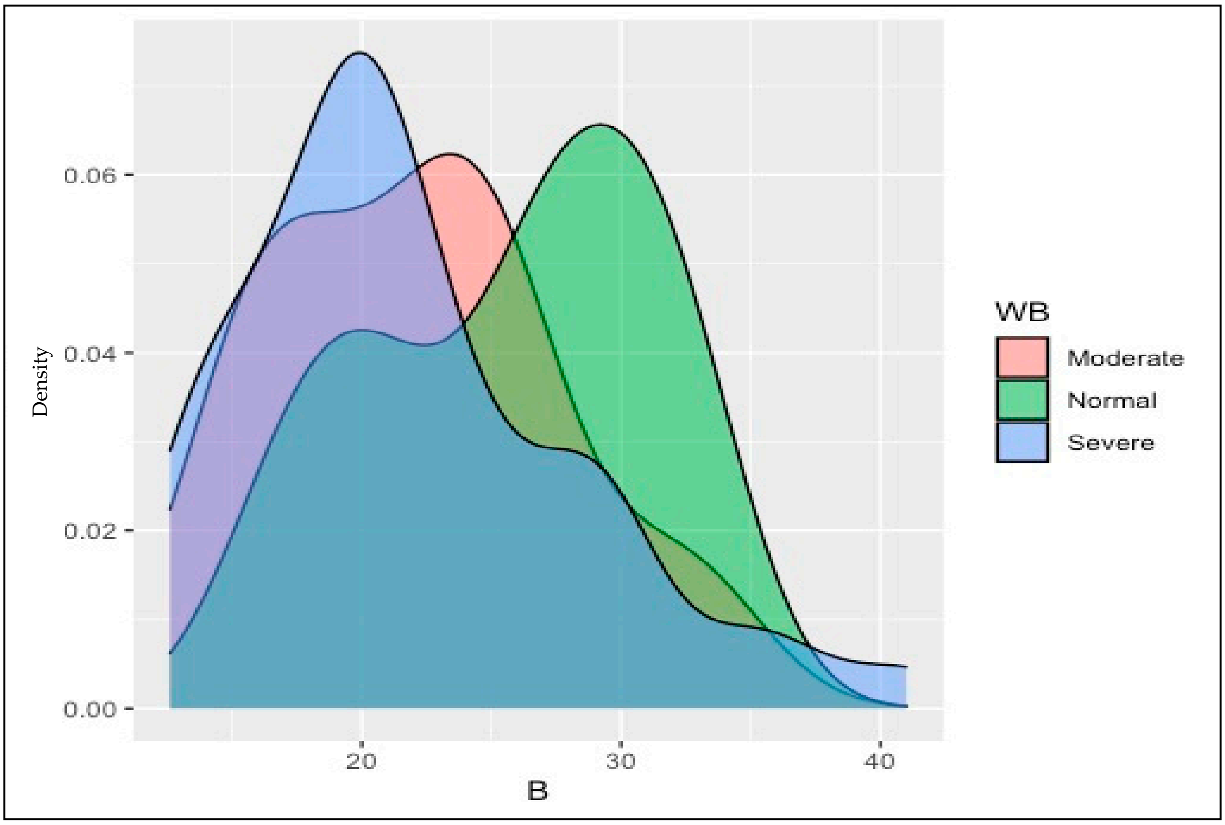

Figure S8. Density graph representing average distribution and overlapping of reactance (B) based on different severity levels

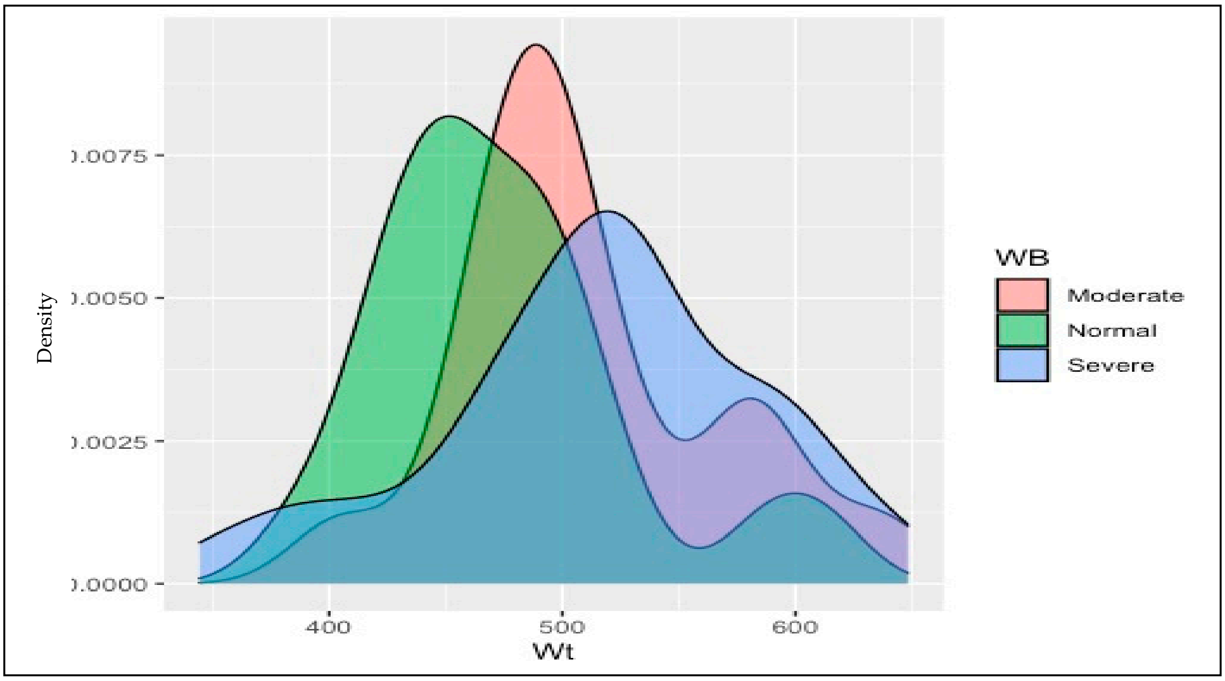

Figure S9. Density graph representing average distribution and overlapping of fillets weights (Wt) based on different severity levels

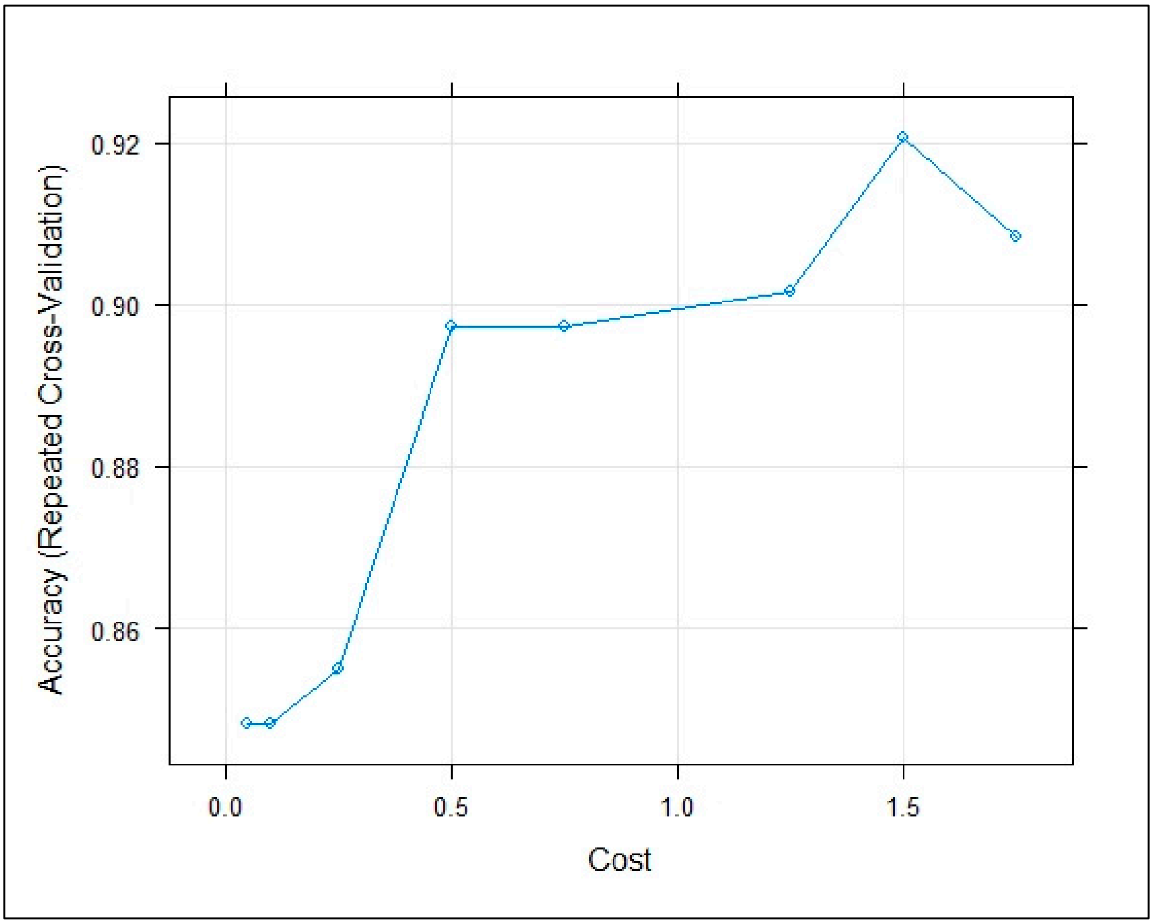

Figure S10. Hand-held BIA collected data graph for repeated cross validation accuracy for SVM model with cost function

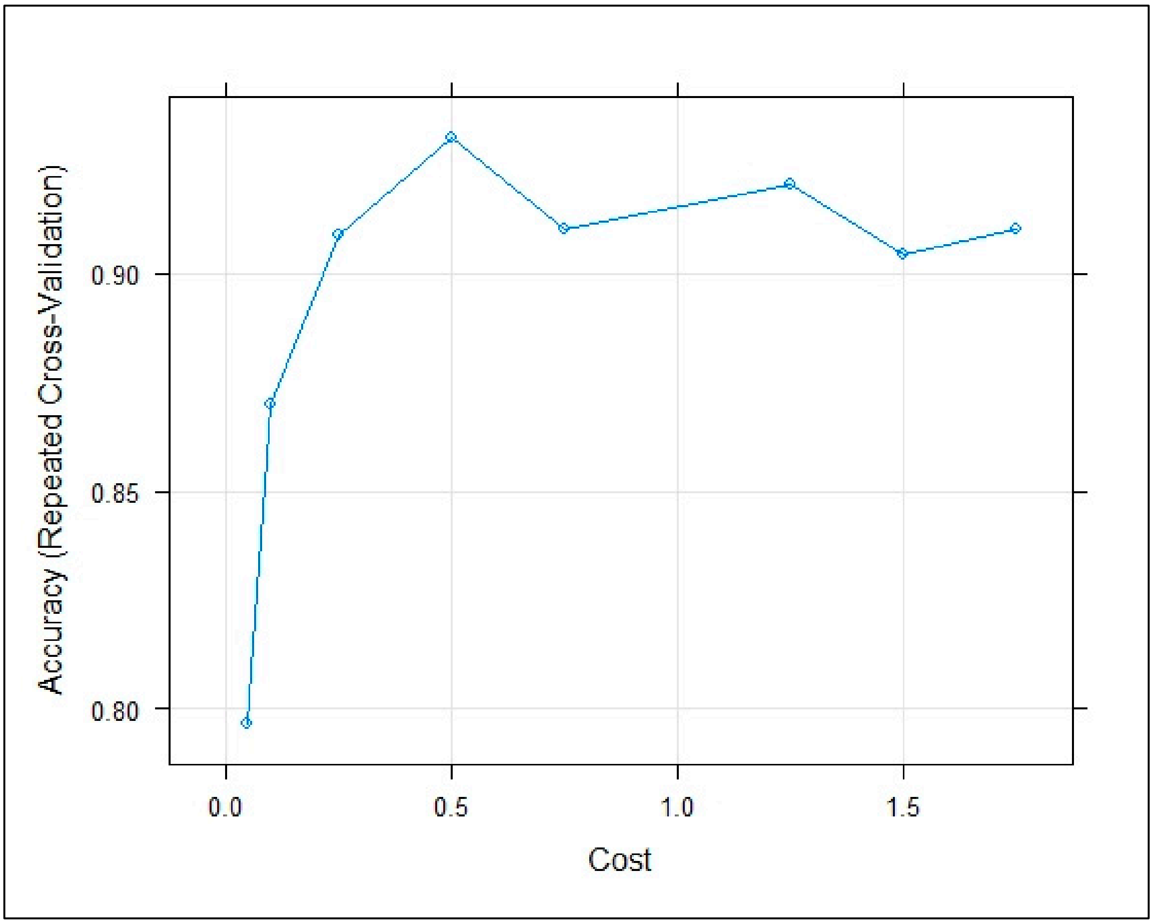

Figure S11. Repeated cross validation accuracy graph for plate BIA collected data in SVM model with cost function
